# A Study on Biocompatible Polymer-Based Packaging of Neural Interface for Chronic Implantation

**DOI:** 10.3390/mi13040516

**Published:** 2022-03-26

**Authors:** HyungDal Park, Wonsuk Choi, Seonghwan Oh, Yong-Jun Kim, Seonho Seok, Jinseok Kim

**Affiliations:** 1 Center for Bionics, Korea Institute of Science and Technology (KIST), Seoul 02792, Korea; hyungdal@kist.re.kr (H.P.); wonsuk@kist.re.kr (W.C.); emart11@kist.re.kr (S.O.); 2 School of Mechanical Engineering, Yonsei University, Seoul 03722, Korea; 3 Department of Biomedical Engineering, Korea University, Seoul 02841, Korea; 4 Center for Nanoscience and Nanotechnology (C2N), University-Paris-Saclay, 91400 Orsay, France

**Keywords:** polymer packaging, neural interface, chronic implantation

## Abstract

This paper proposed and verified the use of polymer-based packaging to implement the chronic implantation of neural interfaces using a combination of a commercial thermal epoxy and a thin parylene film. The packaging’s characteristics and the performance of the vulnerable interface between the thermal epoxy layer and polyimide layer, which is mainly used for neural electrodes and an FPCB, were evaluated through in vitro, in vivo, and acceleration experiments. The performance of neural interfaces—composed of the combination of the thermal epoxy and thin parylene film deposition as encapsulation packaging—was evaluated by using signal acquisition experiments based on artificial stimulation signal transmissions through in vitro and in vivo experiments. It has been found that, when commercial thermal epoxy normally cured at room temperature was cured at higher temperatures of 45 °C and 65 °C, not only is its lifetime increased with about twice the room-temperature-based curing conditions but also an interfacial adhesion is higher with more than twice the room-temperature-based curing conditions. In addition, through in vivo experiments using rats, it was confirmed that bodily fluids did not flow into the interface between the thermal epoxy and FPCB for up to 18 months, and it was verified that the rats maintained healthy conditions without occurring an immune response in the body to the thin parylene film deposition on the packaging’s surface.

## 1. Introduction

Until recently, implantable bioelectronic devices have been developed in various forms for the purpose of monitoring the conditions of diseases through measurements of biological or neural signals and treating or suppressing specific diseases through electrical/optical/magnetic/ultrasound-based stimulations. Basically, implantable bioelectronic devices have been packaged with a material that can minimize the occurrence of an immune response without being damaged or leaking, even when surrounding muscles move after implantations. Most recently, commercialized bioelectronic medical devices have used Ti packaging that can minimize deformations from external forces, such as muscle movements in the main body, and the outside of such packaging is encapsulated with a silicone elastomer that satisfies biocompatibility. At the same time, the packaging has a small number of electrical channels which have millimeter-level diameters, so an individually sealed connector is used to connect it to the main body of implantable bioelectronic devices [1,2,3]. In addition, since most Ti packaging has relatively bulky dimensions compared to neural electrodes, the implantable site is limited to the subcutaneous area of the chest. For the above reason, the neural electrode and the Ti-packaged main body are connected through a wire while maintaining a certain distance or more. Such a configuration does not cause serious problems when the number of channels for neural signal monitoring or stimulation signal transmission is small. However, if the number of channels increases, there is a high possibility of problems due to the plurality of lines connected between the neural electrodes and the main body. In order to prevent failures or the twisting of each wire, a method of winding each line in a circle several times in the body is also applied [4,5]. However, this approach is difficult to utilize as an ultimate solution because it has no choice but to increase the length of the wire inserted into the body and increases the area of the location where the immune response in the body occurs. Recently, demand for a multi-channel neural interface with a high neural signal selectivity and fine local stimulation has increased remarkably. This multi-channel neural interface is being used as an advanced approach to implementing a neural signal-based neuroprosthesis for the daily life recovery of amputees and for electroceuticals, which is rising as a new treatment technology for chronic diseases [6,7,8,9,10,11,12,13,14]. The multi-channel neural interfaces basically have diverse designs depending on the implant sites (brain, spinal cord, peripheral nerve, and vagus nerve), and each neural electrode is commonly connected to a pre-amplifier device regardless of whether it is wire- or wireless-driven [11,12,13,14]. For the connection between the neural electrode and the pre-amplifier device, it is common to use wire bonding or an unsealed connector integrated on a PCB and to mainly use the form of encapsulating the connection part and pre-amplifier device with a biocompatible polymer [15,16,17,18,19]. However, the reason to use the small packaging configuration with the biocompatible polymer can be inferred from the spatial limitation of the implantation site. Moreover, in clinical trials, the system is generally removed again with a surgical procedure after a certain period of an experiment [20,21,22]. Not only has a finite-element analysis study been based on the characteristics of polymer materials applied to polymer-based packaging and materials used as a diffusion barrier but also a verification study of a lifetime has been reported based on a bodily fluid penetration between the polymer insulation layer and the metal electrodes [23,24]. Consequentially, research on polymer-based packaging methods and characteristic verifications are insufficient, even though the purpose of the neural interface is the capability of chronic implantation with the continuous monitoring of the disease condition and delivering stimulation signals.

**Figure 1:**
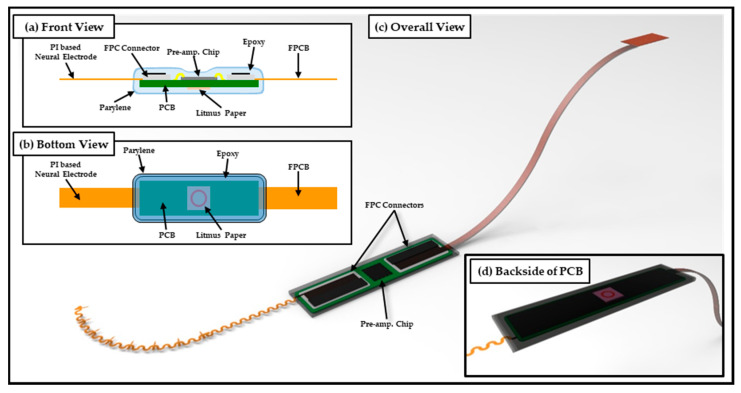
Scheme of polymer-based packaging for neural interface system. (**a**) Front view and (**b**) bottom view show details of packaging inside, (**c**) overall view, and (**d**) backside view.

As shown in Figure 1, in this study, we introduce the biocompatible polymer-based packaging approach for chronic implantation with the results of mechanical property evaluations and acceleration experiments between two kinds of polymers (packages and neural interface materials). Finally, verification results of the lifetime of actually developed polymer-based packaging are discussed with the results of in vivo experiments.

## 2. Materials and Methods

### 2.1. Design and Fabrication of Polymer-Based Neural Interface Packaging Prototype

The neural interface proposed in this study used SPAE (Spiral Probe Array Electrode) [25], a photosensitive polyimide-based neural electrode developed through previous studies; the neural interface system is constructed by connecting PCB integrated with pre-amplifier (RHS2116 Stim/Amplifier Chip, Intan Technologies, Los Angeles, CA, USA) and FPCB (Flexible Printed Circuit Board).

As shown in Figure 1, the proposed neural interface system was encapsulated using thermal epoxy (EPO-TEK^®^ 302, Epoxy Technology, Billerica, MA, USA) from the neural electrode interface to the FPCB interface to connect the PCB substrate and the external system. During the process, square-shaped litmus paper (KA.22-93A, DOOSAN Scientific, Seoul, Korea) responsive to bodily fluids was added to the backside of the PCB in order to enable an intuitive visual leakage check. Thereafter, a thin parylene layer was deposited using commercial parylene deposition equipment (VPC-500, Paco Engineering, Incheon, Korea) to satisfy the biocompatibility of the outer surface of the packaging.

**Figure 2:**
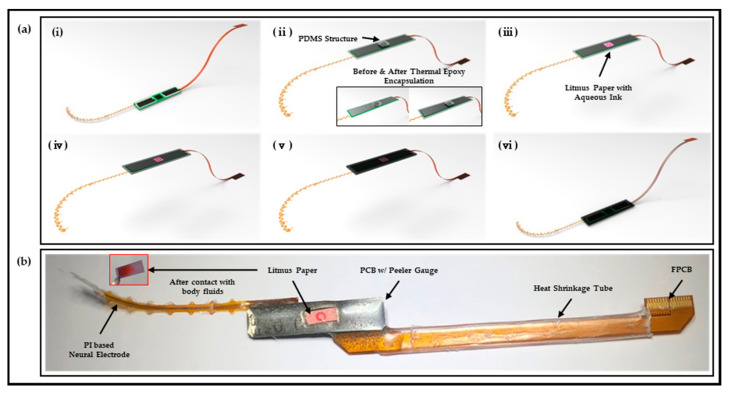
Packaging sample preparation. (**a**) Fabrication process of neural interface system with polymer-based packaging and (**b**) result of sample preparation.

The detailed process of polymer-based packaging of the neural interface is shown in Figure 2a. The fabricated neural electrode and FPCB are individually connected with the PCB through FPC connectors (Molex 502078-3310 and Molex 502078-3760, Molex, Lisle, IL, USA), and the two FPC connectors are electrically connected to the pre-amplifier integrated on the PCB for each channel. In addition, a peeler gauge with a thickness of 50 μm was attached to the backside of the PCB for the convenience of visually checking leakage through litmus paper without any difficulty caused by the contrasting effect of red and green complementary colors (Figure 2a(i)). After integrating each component, encapsulation was performed using thermal epoxy in a state in which a rectangular parallelepiped PDMS (Polydimethylsiloxane, Dow Corning, Midland, MI, USA) structure, which is manufactured with a mixture ratio of 10:1 and a curing condition of 65 °C/1 h and was attached to the back of a PCB (Figure 2a(ii)). The recommendation of the curing condition of thermal epoxy by the manufacturer was 2 h at room temperature. However, it is possible to improve the adhesion between the thermal epoxy and polyimide by adjusting the curing conditions. It should be noted that all thermal epoxy curing processes have been done at 65 °C for 2 h. (Related information can be found in the Results in Section 3.2 and Section 3.3) PDMS has low mechanical strength and adhesion due to weak interactions between molecular chains and a lack of polar groups [26]. Various methods, such as phosphorus and boron synthesis, ultraviolet-ozone treatment, and an oxygen plasma treatment for improving adhesion, have been studied [27,28,29], but in this study, it is possible to easily remove the PDMS structure after curing the thermal epoxy due to the low adhesion of PDMS. Thereafter, square-shaped litmus paper with a circle pattern with an aqueous marker was attached at the corresponding position, and additional encapsulation was carried out using thermal epoxy (Figure 2a(iii,iv)). The entire thickness of the applied thermal epoxy was approximately 2 mm, and the electrode sites of the neural electrode and the metal electrode of the FPCB for electrical connection were covered with a stretchable polymer film (Parafilm, Bemis Company, Inc., Neenah, WI, USA), and a parylene layer of thickness of 5 μm was deposited using a commercial parylene deposition instrument (Figure 2a(v)). After parylene deposition, the stretchable polymer film was removed and the mechanical rigidity was reinforced on FPCB using a heat-shrink tube to complete the manufacturing of the prototype as shown in Figure 2a(vi),b.

### 2.2. Electrophysiological Experiment Setups

**Figure 3:**
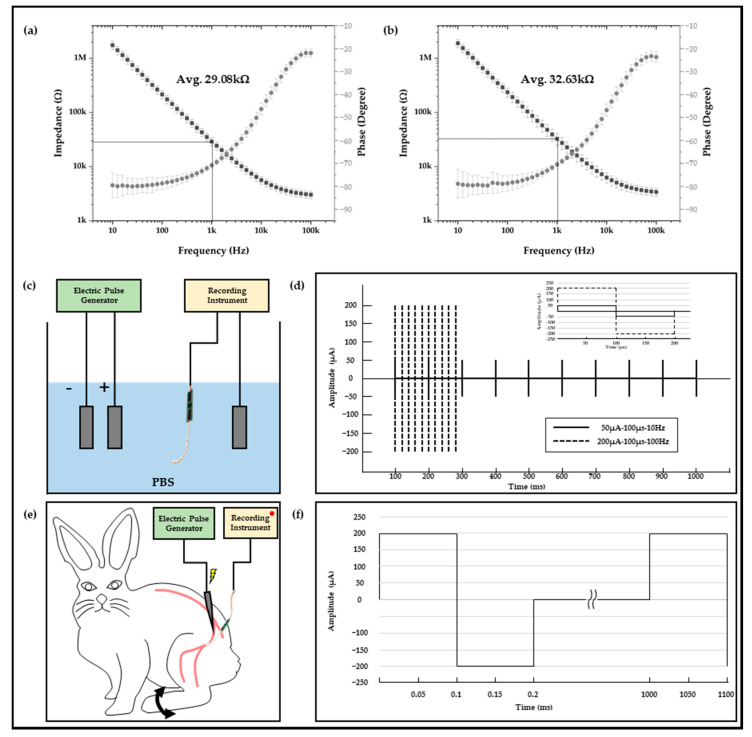
The results of impedance measurements of (**a**) neural electrode for in vitro experiment and (**b**) neural electrode for in vivo experiment. Electrophysiological experiment setup and stimulation parameters. (**c**) In vitro experiment setup in PBS, (**d**) a couple of stimulation parameters, (**e**) in vivo experiment setup with New Zealand white rabbit, and (**f**) in vivo stimulation parameters.

In vitro and in vivo experiments were conducted to verify whether there was a sufficiently applicable performance of the neural interface to which the polymer-based packaging was applied or not. In order to verify the signal acquisition performances of the neural interfaces to be used before the relevant experiment, the impedance at each frequency was measured using electrochemical impedance spectroscopy. The impedances of a total of two neural interfaces were measured, and each measurement result exhibited an average 29.08 kOhm and 32.63 kOhm at 1 kHz. This is a sufficient impedance value suitable for acquiring a neural signal, and the standard deviation of each average impedance was simultaneously analyzed to be 5.02 kOhm and 8.17 kOhm; thus, it was also verified that it had stability. In addition, the neural interface obtained from the measurement result of Figure 3a was used for in vitro experiment, and the neural interface with the impedance of Figure 3b was used for in vivo experiment, respectively. As shown in Figure 3c, the in vitro experiments were configured in a state in which the anode/cathode electrodes, which were connected to the electric pulse generator (HSE-HA stimulator CS for isolated cells, Hugo Sachs Elektronik Harvard Apparatus GmbH, Baden-Württemberg, Germany), and the neural interface and reference electrode, which were connected to the neural signal acquisition equipment (RHS stim/recording system, Intan Technologies, Los Angeles, CA, USA), were immersed in a PBS solution. This experiment was a process to verify the performance and capability of the neural interface that acquired the artificial signal generated through an electric pulse generator, and the stimulation parameter was constructed based on the criteria of the general neural stimulation parameter [30,31,32]. As shown in Figure 3d, the stimulus parameters were composed of two types (50 μA-100 μs-10 Hz and 200 μA-100 μs-100 Hz). In addition, the corresponding stimulation signal generated by the electrical pulse generator was acquired and recorded in real time from a total of 32 channel electrodes using a neural signal acquisition device.

### 2.3. In Vivo Experiment Setups

In addition to verification through the in vitro experiment, the in vivo experiments using the New Zealand male white rabbit was configured as shown in Figure 3e. The stimulation probe was connected to the electric pulse generator to apply stimulation, and the neural electrode applied to the neural interface was implanted into the sciatic nerve of the rabbit for signal acquisition. Implantation of the neural electrode was performed using a surgical method established using rats in previous study [25], and a 12-week-old New Zealand male white rabbit was anesthetized through laparoscopic injection of a Zoletil (Virbac, Carros, France)–Rompun (BAYER, Leverkusen, Germany) with composition ratio of 3:1. After anesthetizing, the neural electrode was inserted into the sciatic nerve, which was exposed through a muscle incision. The verification experiment was conducted under breathing anesthesia, and the stimulation signal interval was set to 1000 ms so that the movement of the leg of the rabbit by the sciatic nerve stimulation could be visually classified. Figure 3e,f show the configuration and results of the in vivo experiment, for which a system capable of acquiring and recording stimulation signals in real time using neural signal acquisition equipment was constructed. All procedures for animal testing were performed through the Institutional Animal Care and Use Committee (IACUC) guidelines of the Korea Institute of Science and Technology (KIST).

### 2.4. Test Sample Preparation for Polyimide (FPCB)-Epoxy Peel-Off Test

**Figure 4:**
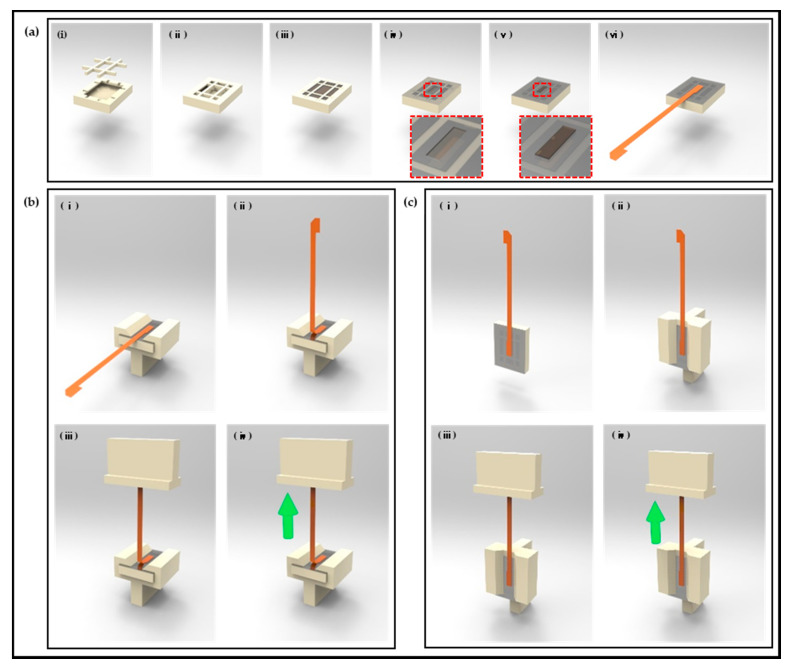
Procedures of adhesion evaluation test sample preparation. (**a**) Process of sample preparation with uniform adhered area between epoxy and FPBC; (**a-i**–**a-ii**) preparation and assembly of 3D-printed sample holder; (**a-iii**) filling and curing epoxy into vacancy of sample holder; (**a-iv**) covering epoxy surface with detachable tape excluding uniform area; (**a-v**) additional epoxy filling and squeezing epoxy into uncovered area; and (**a-vi**) applying FPCB to uncured epoxy and curing. (**b**) Tear-test procedures. (**c**) Shear-test procedures.

The polymer-based packaging for neural interface in this study was mainly configured using thermal epoxy encapsulation. After thermal epoxy encapsulation, thin parylene film, which is widely used as a coating material to prevent immune reactions and achieve biocompatibility, was deposited. [33,34,35] In this configuration, thermal epoxy was applied as a major material for polymer-based packaging, and the adhesion between thermal epoxy and the material forming the neural interface is directly related to the packaging’s life and performance. Because the interface is bound to form between thermal epoxy and polyimide, which is the main component of the FPCB applied for electrical connection with the neural interface and external system, this interface in packaging is the most vulnerable site. Accordingly, samples for experiments were manufactured in the order shown in Figure 4 to verify the adhesion between the thermal epoxy and polyimide depending on the curing conditions of the thermal epoxy. The experiment was constructed in a form that could apply tear force and shear force by attaching a specific area of thermal epoxy and FPCB. First, a sample with a specific area of thermal epoxy and FPCB was manufactured as shown in Figure 4a. A structure capable of containing a certain amount of thermal epoxy and a #-shaped structure for preventing the separation of the thermal epoxy from the sample structure during the adhesion measurement experiment were printed by using a commercial 3D printer (CUBICON Single Plus-320C, Cubicon, Seongnam, Korea) and were manually assembled (Figure 4a(i,ii)). Thereafter, thermal epoxy was poured into the structure and cured at room temperature for 2 h as per recommended curing condition of the manufacturer (Figure 4a(iv)), and the thermal epoxy surface was covered except for a pre-determined area with a commercial detachable tape (Scotch^®^ Magic™ tape, 3M, Saint Paul, MN, USA; Figure 4a(v)). The exposed surface area of the thermal epoxy is established by 1 mm offset from the outer line of the FPCB to the inside. This offset was set to prevent the applied thermal epoxy from being attached to the outer line or edge of the FPCB and acting as an obstacle to adhesion measurement of the interface. To specify a volume of the second thermal epoxy through the thickness (about 60 μm) of commercial detachable tape, the identical thermal epoxy was spread to the exposed surface and the squeezing method was carried out in stencil printing using a peeler gauge with a thickness of 100 μm (Figure 4a(v)) [36]. In the final step shown in Figure 4a(vi), the second thermal epoxy curing was performed with an FPCB placed thereon and a weight of 3 kg placed on top for conformal attachment. Though different products from the thermal epoxy were used in this research, the curing condition was established based on research results that when the thermal epoxy, for which studies recommend a room-temperature curing condition, is cured at a higher temperature, its curing rigidity can be increased [37]. The thermal epoxy of the sample applied in the experiment was cured at 45 °C/2h and 65 °C/2 h, respectively, which were higher than room temperature.

### 2.5. Peel-Off Test Setup for Polyimide (FPCB) Epoxy

Additional structures capable of applying tear force and shear force for the measurement of adhesion between thermal epoxy and polyimide were configured as shown in Figure 4b,c. A structure for fixing the FPCB thermal epoxy-attached sample to the chuck of the tensile experimental equipment and a structure that can be grasped the end of the FPCB by another chuck of the tensile experimental equipment were output using a 3D printer. The structure for fixing the FPCB thermal epoxy-attached sample was configured to have a difference of 90 degree angle to enable tear force and shear force applications, respectively. In the experiment for evaluating the adhesion between thermal epoxy and polyimide (FPCB) using the prepared sample, commercial tensile experimental equipment (Shimadzu EZ-S machine, Shimadzu, Kyoto, Japan) was used and tensile stroke was applied to one end of the FPCB under conditions of 1 mm/min; the generated force was measured in mN units.

### 2.6. Acceleration Test Setup

The reliabilities of acceleration experiments using temperature applications higher than the used temperature are generally derived using the Arrhenius equation as shown below in Equation (1).

(1) $$A_{T}=\frac{\lambda_{T1}}{\lambda_{T2}}=\exp\left[\left(\frac{-E_{a}}{k}\right)\left(\frac{1}{T_{1}}-\frac{1}{T_{2}}\right)\right]$$

where $E_{a}$ is the activation energy (eV), k is Boltzmann’s constant as $8.62\times 10^{-5}\mathrm{eV}/K$, $T_{1}$ is the absolute temperature of application condition, $T_{2}$ is the absolute temperature of experiment condition, $\lambda_{T1}$ is the observed failure rate at application condition, and $\lambda_{T2}$ is the observed failure rate at experiment condition. Although the life of polymer-based packaging according to the acceleration experiment can be predicted based on the Arrhenius equation, an acceleration experiment was constructed on the basis that about 100 days of lifetime verification with 300 h of aging can be performed on an environment 30 °C higher than the usage temperature based on Donaldson’s report [38]. Three samples (a total of 24 samples, 12 45 °C cured samples and 12 65 °C cured samples) were each taken out at 50, 100, 200, and 300 h of aging while immersed in a 65 °C PBS solution, and then tear force was applied to evaluate the changes in interfacial adhesion between the thermal epoxy and FPCB.

### 2.7. In Vivo Chronic Implantation

To verify the practical applicability of fabricated neural interfaces to which polymer-based packaging was applied, in vivo experiments were constructed. Neural electrodes were inserted into rats’ sciatic nerves, and sites, including PCB and FPCB, were placed in the rats’ hind leg muscles and subcutaneously. Insertion surgeries were performed on a total of two individual rats, and the electrodes were extracted at the times of 8 months and 18 months after implantation to check for changes in the conditions of the overall polymer-based packaging, especially applied litmus papers. At the time of extractions, it was difficult to separate the neural electrodes from the sciatic nerves, so the neural electrodes were cut and extracted.

## 3. Results

### 3.1. In Vitro and In Vivo Characterizations of Polymer-Based Neural Interface Packaging Prototype

**Figure 5:**
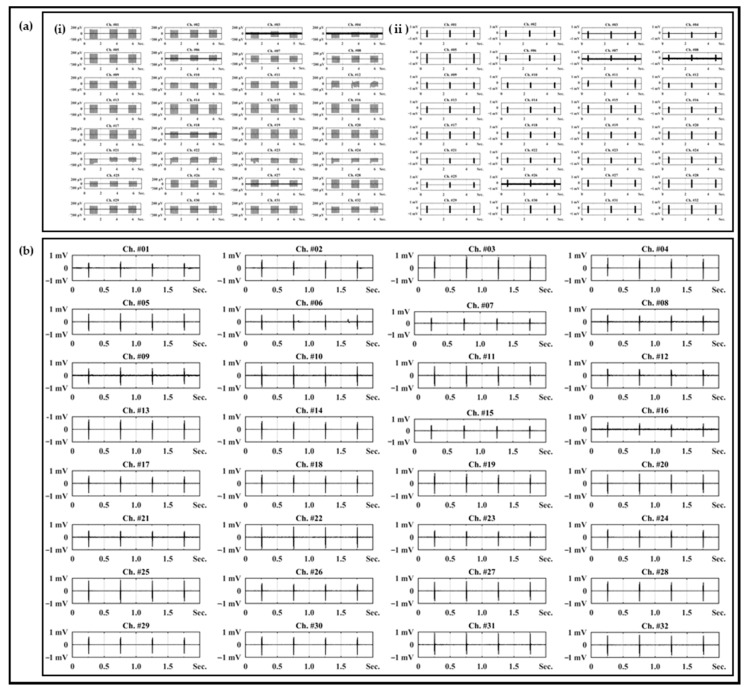
Results of in vitro and acute in vivo experiments. (**a**) Recording results in PBS in vitro experiment with different biphasic stimulation parameters (50 μA-100 μs-10 Hz and 200 μA-100 μs-100 Hz) and (**b**) in vivo recording results in sciatic nerve of New Zealand white rabbit with biphasic stimulation parameter (200 μA-100 μs-1 Hz).

In the PBS solution-based in vitro experiment, two artificial stimulation signals (50 μA-100 μs-10 Hz and 200 μA-100 μs-100 Hz) were applied, and the results acquired using 32 electrode channels formed in the neural electrode and commercial neural signal acquisition equipment are shown in Figure 5a. For each artificial stimulation signal, it can be evaluated that signals similar to stimulation signals were acquired from all 32 channels. Similarly, for in the in vivo experiment using a New Zealand male white rabbit, it was confirmed that stimulation signals of 200 μA-100 μs-1 Hz could be obtained from all 32 channels (Figure 5a). In addition, the relevant procedures and results of the in vivo experiment can be confirmed through Video S1, and the contraction of the leg muscles of the rabbit with a certain cycle by a stimulation signal can be clearly verified. Through in vitro and in vivo experiments, it was confirmed that the neural interface to which the polymer-based packaging was applied operated as an initial purpose. In addition, a quantitative analysis of the signals acquired through the experiments was carried out, and the results are summarized in Table 1. The maximum, minimum, average, and standard deviation values were derived from the positive and negative regions of the acquisition signals for a total of three stimulation conditions. Also, the average and standard deviation values of the entire signal amplitude were also calculated. The difference between the maximum and minimum values in each region was up to 342.0 μV under 200 μA-100 μs-10 Hz stimulation conditions, but the standard deviation of the region was 89.7 μV. Even under other stimulation conditions, the standard deviation in the positive and negative regions was from a minimum of 26.5 μV to a maximum of 90.8 μV. In a process of acquiring a signal using a neural electrode, the deviation may sufficiently occur depending on system parameters, such as electrode impedance, cross-talk between electrodes, and unknown recording conditions, and the result of impedance measurement research is reported to be different over time in the same system configuration [39]. In comparison, although the maximum/minimum value of the total acquisition signal differs, it cannot be determined that the non-uniform signal was obtained on a single electrode itself because the average and standard deviation of the total signal size specified in Table 1 are not abnormally large. Accordingly, it is determined that every acquired signal amplitude is a result of different distances from the stimulation signal-generation position, the difference in impedance for each electrode, and the cross-talk between electrodes. As a result, it can be evaluated that a sufficient neural signal acquisition performance and post-processing, such as clustering for acquired neural signal analyses, have sufficiently possible signal levels.

### 3.2. Adhesion Evaluation of Peel-Off Test

**Figure 6:**
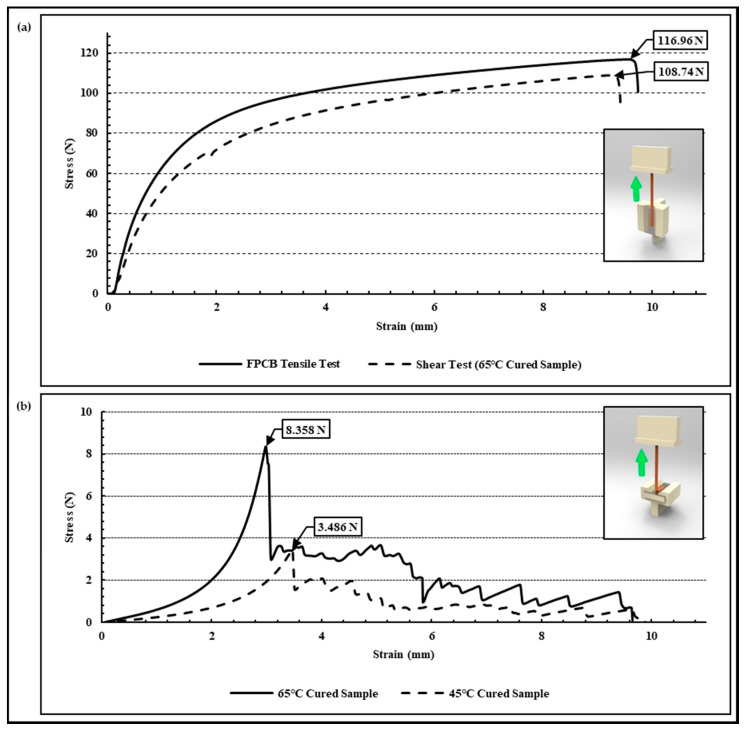
Results of Adhesion test. (**a**) Shear-force induced samples and (**b**) tear-force induced samples.

The results of the tear force and shear force experiments conducted for the evaluation of the interface adhesion between the thermal epoxy and FPCB are as shown in Figure 6. When shear force is applied to a sample manufactured under a curing condition of 65 °C/2 h, the FPCB is not separated from the thermal epoxy; rather, it fractures at the middle point of the FPCB. As shown in the graph of Figure 6a, the measurement results of the shear forces are similar or higher to the tensile strength of the FPCB itself. It means that the sheer force experiment is not suitable for the evaluation of the interfacial adhesion between the thermal epoxy and the FPCB (polyimide) because the FPCB can be deformed or fractured before detaching it. Conversely, in the tear force experiment, it was verified that the FPCB was separated from the thermal epoxy based on 3.486 N and 8.358 N, respectively, in the samples prepared under curing conditions of 45 °C/2 h and 65 °C/2 h. As a result, in the situation with the same physically adhered interface conditions, it was evaluated that the interfacial adhesion differed more than twice in accordance with the curing conditions of the thermosetting epoxy.

### 3.3. Lifetime and Characteristics of Acceleration Test

**Figure 7:**
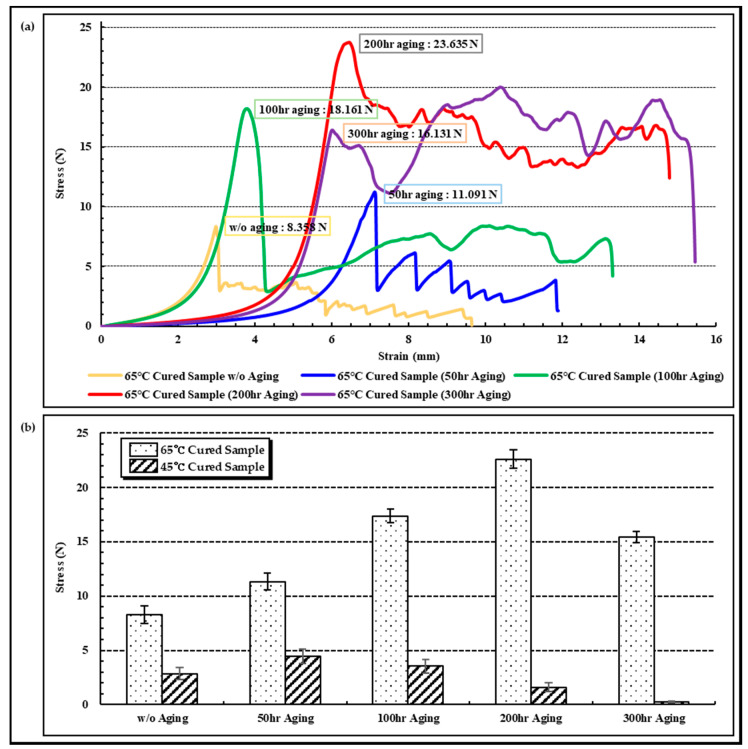
Results of acceleration aging test in 65 °C PBS solution at 50, 100, 200, 300 h. (**a**) Results of tear test of 65 °C cured sample after each aging time and (**b**) maximum tear force comparison between 45 °C and 65 °C cured samples.

In an acceleration experiment conducted to confirm the changes in interfacial adhesion according to the curing conditions, a tear force application experiment was conducted on the samples, which were exposed to a 65 °C PBS solution for 50, 100, 200, and 300 h aging times. The interfacial adhesion characteristics of the samples, which were prepared under curing conditions of 65 °C/2 h, are expressed for each aging time period in Figure 7a. It can be confirmed that the interfacial adhesion increases rather than the results of the initial tear force experiment until 50 h, 100 h, and 200 h elapse. This means that although the room-temperature-curable thermal epoxy used in the experiment was cured under a higher temperature at 65 °C/2 h curing conditions, a full curing was not achieved. In this regard, it can be determined through the results of Lapique’s research [37] that show a difference in the degree of full curing depending not only on the curing temperature but also on the curing time for the room-temperature-curable thermal epoxy. Although it can be seen that the interfacial adhesion decreased to about 32% when 300 h had elapsed compared to when 200 h had elapsed, based on the situation where the 300 h elapsed point was maintained at a higher level than the initial interface adhesion, it can be predicted that the interface between the thermal epoxy and FPCB maintains its performance. The acceleration experiment results for the samples manufactured under 45 °C/2 h and 65 °C/2 h curing conditions were summarized in Figure 7b, and the samples with 45 °C/2 h curing conditions showed a tendency to increase interfacial adhesion until 50 h but decreased from 100 h and were lower than the initial value at 200 h. Assuming that the body’s temperature was 35 °C, it can be predicted that the lifetime of the sample manufactured under the 45 °C/2 h curing condition does not exceed 2 months and that the sample with the 65 °C/2 h curing condition has a lifespan of 100 days or more.

### 3.4. In Vivo Chronic Implantation

**Figure 8:**
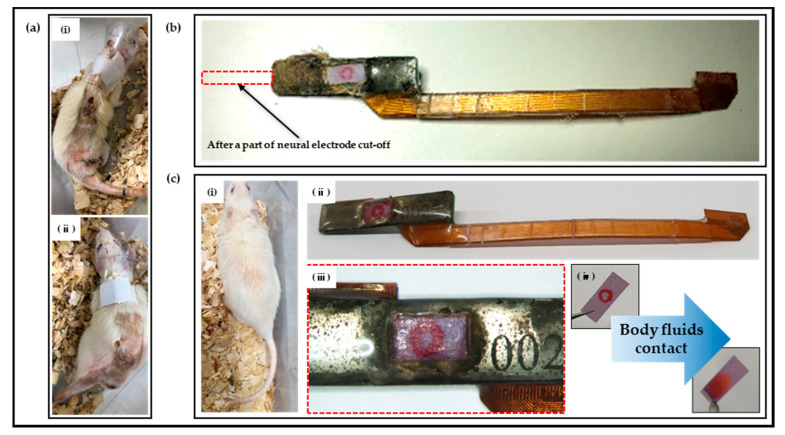
Results of in vivo experiments. (**a**) Two rats after packaged neural electrode implantations, (**b**) photograph of neural interface extracted after 8 months of implantation, (**c-i**) a rat of 18 months after implantation (its hair grew back in the surgical area and maintained a healthy state), (**c-ii**) photograph of neural interface extracted after 18 months of implantation, (**c-iii**) no discoloration of litmus paper and diffusion of aqueous ink, and (**c-iv**) an example of discoloration of litmus paper and diffusion of aqueous ink with bodily fluid contact.

As shown in Figure 8, the polymer-based packaged neural interfaces, which were inserted into the sciatic nerves of, fixed to the muscles of, and fixed subcutaneously to rats, were extracted at the times of 8 months (Figure 8b) and 18 months (Figure 8c) after their insertions to confirm the discoloration and circle-shaped aqueous ink smudging of the litmus papers, respectively. Since the discoloration of the litmus papers and the diffusion of the aqueous ink were not confirmed, it was verified that bodily fluids were not flowing into the interface between the thermal epoxy and the FPCB interface formed in the polymer-based packaging; additionally, the polymer-based packaging was not deformed or damaged even from the hind limb movements of the rats. It was additionally confirmed that the rats were in healthy conditions, which could be estimated based on their behavior, the average amounts of daily intake 18 months after the implantations, and the fact that inflammation caused by the immune response had not formed around the implanted neural interfaces due to the thin parylene film coating for biocompatibility.

## 5. Conclusions

To achieve the ultimate purpose of the neural interface, a polymer-based packaging approach to chronic implantations that was capable of biological monitoring and applications of various stimulation signals was proposed. The possibility of chronic implantation was deduced through mechanical property evaluations, acceleration experiments, and in vitro and in vivo experiments. In addition, it was confirmed that when applying commercial thermal epoxy to polymer-based packaging, it is necessary to establish a full curing condition for each, not a curing condition proposed by a product manufacturer. Due to the realistic limitation that the existing neural interface does not operate normally over 18 months, it was necessary to verify the packaging performance by examining for a discoloration of litmus paper and a spreading of aqueous ink. However, it was found that the life of the proposed polymer-based packaging was more than 18 months as bodily fluids did not penetrate the interface between the thermal epoxy and the polyimide through as confirmed by examining changes in litmus paper. The packaging technology verified through this study can be used as a novel polymer-based packaging method for the integration of neural electrodes and pre-amplifiers for implementing neural interfaces with various purposes, such as neural signal monitoring or neural stimulation. The packaging method is consequently expected to improve the possibility of chronic implantations of neural interfaces. Furthermore, analyses of interfacial properties and the optimization of application conditions between all the materials used in the packaging including the proposed packaging combination have to accompany for reliable polymer-based packaging accomplishment.

## Figures and Tables

**Table 1:** Comparison of the characteristics of signals acquired through in vitro and in vivo experiments.

Samples	Stimulation Parameter	Signal Amplitude (μV)									
		Positive Max.	Positive Min.	Negative Max.	Negative Min.	Positive Avg.	Positive Std. Dev.	Negative Avg.	Negative Std. Dev.	Total Amplitude Avg.	Total Amplitude Std. Dev.
**In vitro sample**	50 μA-100 μs-10 Hz	183.3	15.3	−63.9	−189.8	128.3	50.8	−165.5	26.5	293.8	54.0
	200 μA-100 μs-10 Hz	896.5	554.5	−592.0	−897.6	641.7	89.7	−816.2	90.8	1457.6	123.0
**In vivo sample**	200 μA-100 μs-1 Hz	884.8	650.8	−811.8	−899.6	797.9	80.8	−848.2	33.9	1646.0	107.7

## Supplementary Materials

The following supporting information can be downloaded at: https://www.mdpi.com/article/10.3390/mi13040516/s1. Video S1: Stimulation & Recording Acute in-vivo Experiment.

## References

[1] Patschger A., Hopf A., Güpner M., Bliedtner J. (2016). Laser Material Processing of Medical Titanium: Titanium boxes for hermetic transport of medical implants. Laser Tech. J..

[2] Yang H., Wu T., Zhao S., Xiong S., Peng B., Humayun M.S. Chronically implantable package based on alumina ceramics and titanium with high-density feedthroughs for medical implants. Proceedings of the 2018 40th Annual International Conference of the IEEE Engineering in Medicine and Biology Society (EMBC).

[3] Jiang G., Zhou D.D. (2009). Technology advances and challenges in hermetic packaging for implantable medical devices. Implantable Neural Prostheses 2.

[4] Dewald H.A., Lukyanenko P., Lambrecht J.M., Anderson J.R., Tyler D.J., Kirsch R.F., Williams M.R. (2019). Stable, three degree-of-freedom myoelectric prosthetic control via chronic bipolar intramuscular electrodes: A case study. J. Neuroeng. Rehabil..

[5] Pope J.E., Carlson J.D., Rosenberg W.S., Slavin K.V., Deer T.R. (2016). Peripheral nerve stimulation for pain in extremities: An update. Stimul. Peripher. Nerv. Syst..

[6] Tan D.W., Schiefer M.A., Keith M.W., Anderson J.R., Tyler J., Tyler D.J. (2014). A neural interface provides long-term stable natural touch perception. Sci. Transl. Med..

[7] Oddo C.M., Raspopovic S., Artoni F., Mazzoni A., Spigler G., Petrini F., Micera S. (2016). Intraneural stimulation elicits discrimination of textural features by artificial fingertip in intact and amputee humans. eLife.

[8] Koopman F.A., Schuurman P.R., Vervoordeldonk M.J., Tak P.P. (2014). Vagus nerve stimulation: A new bioelectronics approach to treat rheumatoid arthritis?. Best Pract. Res. Clin. Rheumatol..

[9] Haidar A., Legault L., Messier V., Mitre T.M., Leroux C., Rabasa-Lhoret R. (2015). Comparison of dual-hormone artificial pancreas, single-hormone artificial pancreas, and conventional insulin pump therapy for glycaemic control in patients with type 1 diabetes: An open-label randomised controlled crossover trial. Lancet Diabetes Endocrinol..

[10] Güemes A., Georgiou P. (2018). Review of the role of the nervous system in glucose homoeostasis and future perspectives towards the management of diabetes. Bioelectron. Med..

[11] Sung C., Jeon W., Nam K.S., Kim Y., Butt H., Park S. (2020). Multimaterial and multifunctional neural interfaces: From surface-type and implantable electrodes to fiber-based devices. J. Mater. Chem. B.

[12] Coker R.A., Zellmer E.R., Moran D.W. (2019). Micro-channel sieve electrode for concurrent bidirectional peripheral nerve interface. Part B: Stimulation. J. Neural Eng..

[13] Christensen M.B., Pearce S.M., Ledbetter N.M., Warren D.J., Clark G.A., Tresco P.A. (2014). The foreign body response to the Utah Slant Electrode Array in the cat sciatic nerve. Acta Biomater..

[14] Kundu A., Harreby K.R., Yoshida K., Boretius T., Stieglitz T., Jensen W. (2013). Stimulation selectivity of the “thin-film longitudinal intrafascicular electrode”(tfLIFE) and the “transverse intrafascicular multi-channel electrode”(TIME) in the large nerve animal model. IEEE Trans. Neural Syst. Rehabil. Eng..

[15] Sharma A., Rieth L., Tathireddy P., Harrison R., Oppermann H., Klein M., Solzbacher F. (2011). Long term in vitro functional stability and recording longevity of fully integrated wireless neural interfaces based on the Utah Slant Electrode Array. J. Neural Eng..

[16] Shon A., Chu J.U., Jung J., Kim H., Youn I. (2018). An implantable wireless neural interface system for simultaneous recording and stimulation of peripheral nerve with a single cuff electrode. Sensors.

[17] Kang Y.N., Chou N., Jang J.W., Byun D., Kang H., Moon D.J., Kim S. (2019). An intrafascicular neural interface with enhanced interconnection for recording of peripheral nerve signals. IEEE Trans. Neural Syst. Rehabil. Eng..

[18] Pena A.E., Kuntaegowdanahalli S.S., Abbas J.J., Patrick J., Horch K.W., Jung R. (2017). Mechanical fatigue resistance of an implantable branched lead system for a distributed set of longitudinal intrafascicular electrodes. J. Neural Eng..

[19] Skok T., Tabakow P., Chmielak K. (2019). Methods of integrating the human nervous system with electronic circuits. Adv. Clin. Exp. Med..

[20] Rijnbeek E.H., Eleveld N., Olthuis W. (2018). Update on peripheral nerve electrodes for closed-loop neuroprosthetics. Front. Neurosci..

[21] George J.A., Page D.M., Davis T.S., Duncan C.C., Hutchinson D.T., Rieth L.W., Clark G.A. (2020). Long-term performance of Utah slanted electrode arrays and intramuscular electromyographic leads implanted chronically in human arm nerves and muscles. J. Neural Eng..

[22] Kaijankoski H., Nissen M., Ikäheimo T.M., von Und Zu Fraunberg M., Airaksinen O., Huttunen J. (2019). Effect of spinal cord stimulation on early disability pension in 198 failed back surgery syndrome patients: Case-control study. Neurosurgery.

[23] Seok S. (2021). Polymer-Based Biocompatible Packaging for Implantable Devices: Packaging Method, Materials, and Reliability Simulation. Micromachines.

[24] Jeong J., Bae S.H., Seo J.M., Chung H., Kim S.J. (2016). Long-term evaluation of a liquid crystal polymer (LCP)-based retinal prosthesis. J. Neural Eng..

[25] Kim O., Choi W., Jung W., Jung S., Park H., Jeong J., Chu J.U., Park J.W., Kim J. (2020). Spirally Arrayed Electrode for Spatially Selective and Minimally Displacive Peripheral Nerve Interface. J. Microelectromechanical Syst..

[26] Martin S., Bhushan B. (2017). Transparent, wear-resistant, superhydrophobic and superoleophobic poly (dimethylsiloxane)(PDMS) surfaces. J. Colloid Interface Sci..

[27] Wang Y., Ling Y., Zhou S., Chen Y., Liang M., Zou H. (2021). Enhanced mechanical and adhesive properties of PDMS based on novel PDMS-epoxy IPN structure. J. Polym. Res..

[28] Wang Q., Xiong L., Liang H., Chen L., Huang S. (2018). Synthesis of a novel polysiloxane containing phosphorus, and boron and its effect on flame retardancy, mechanical, and thermal properties of epoxy resin. Polym. Compos..

[29] Amerian M., Amerian M., Sameti M., Seyedjafari E. (2019). Improvement of PDMS surface biocompatibility is limited by the duration of oxygen plasma treatment. J. Biomed. Mater. Res. Part A.

[30] Merrill D.R., Bikson M., Jefferys J.G. (2005). Electrical stimulation of excitable tissue: Design of efficacious and safe protocols. J. Neurosci. Methods.

[31] Newbold C., Richardson R., Millard R., Seligman P., Cowan R., Shepherd R. (2011). Electrical stimulation causes rapid changes in electrode impedance of cell-covered electrodes. J. Neural Eng..

[32] Günter C., Delbeke J., Ortiz-Catalan M. (2019). Safety of long-term electrical peripheral nerve stimulation: Review of the state of the art. J. Neuroeng. Rehabil..

[33] Li J., Kang L., Yu Y., Long Y., Jeffery J.J., Cai W., Wang X. (2018). Study of long-term biocompatibility and bio-safety of implantable nanogenerators. Nano Energy.

[34] Kuo W.C., Wu T.C., Wu C.F., Wang W.C. (2021). Bioperformance analysis of parylene C coating for implanted nickel titanium alloy. Mater. Today Commun..

[35] Iacovacci V., Naselli I., Salgarella A.R., Clemente F., Ricotti L., Cipriani C. (2021). Stability and in vivo safety of gold, titanium nitride and parylene C coatings on NdFeB magnets implanted in muscles towards a new generation of myokinetic prosthetic limbs. RSC Adv..

[36] Rusdi M.S., Abdullah M.Z., Aziz M.S.A., Abdullah M.K., Chellvarajoo S., Husin A., Rethinasamy P., Veerasamy S. (2018). Multiphase flow in solder paste stencil printing process using CFD approach. J. Adv. Res. Fluid Mech. Therm. Sci..

[37] Lapique F., Redford K. (2002). Curing effects on viscosity and mechanical properties of a commercial epoxy resin adhesive. Int. J. Adhes. Adhes..

[38] Donaldson P.E.K., Sayer E. (1981). A technology for implantable hermetic packages. Part 2: An implementation. Med. Biol. Eng. Comput..

[39] Perge J.A., Homer M.L., Malik W.Q., Cash S., Eskandar E., Friehs G., Donoghue J., Hochberg L.R. (2013). Intra-day signal instabilities affect decoding performance in an intracortical neural interface system. J. Neural Eng..

